# Emotion differentiation in early recovery from alcohol use disorder: Associations with in‐the‐moment affect and 3‐month drinking outcomes

**DOI:** 10.1111/acer.14854

**Published:** 2022-05-25

**Authors:** Noah N. Emery, Kyle J. Walters, Lili Njeim, Maya Barr, Daniella Gelman, David Eddie

**Affiliations:** ^1^ 3447 Department of Psychology Colorado State University Fort Collins Colorado USA; ^2^ 2345 Department of Psychiatry and Behavioral Sciences Medical University of South Carolina Charleston South Carolina USA; ^3^ Department of Psychology University of South Dakota Vermillion South Dakota USA; ^4^ 1846 School of Public Health Boston University Boston Massachusetts USA; ^5^ 1812 Department of Psychology Harvard University Cambridge Massachusetts USA; ^6^ Emma Pendleton Bradley Hospital Alpert Medical School Brown University Riverside Rhode Island USA; ^7^ 1811 Recovery Research Institute Center for Addiction Medicine Massachusetts General Hospital Harvard Medical School Boston Massachusetts USA

**Keywords:** alcohol use disorder, emotion, emotion differentiation, recovery, stress

## Abstract

**Background:**

Early recovery from alcohol use disorder (AUD) is commonly associated with high levels of negative affect, stress, and emotional vulnerability, which confer significant relapse risk. Emotion differentiation—the ability to distinguish between discrete emotions—has been shown to predict relapse after treatment for a drug use disorder, but this relationship has not been explored in individuals recovering from AUD.

**Methods:**

The current study used thrice daily random and up to thrice daily self‐initiated ecological momentary assessment surveys (*N* = 42, observations = 915) to examine whether 1) moments of high affective arousal are characterized by momentary differences in emotion differentiation among individuals in the first year of a current AUD recovery attempt, and 2) individuals’ average emotion differentiation would predict subsequent alcohol use measured by the timeline follow‐back over a 3‐month follow‐up period.

**Results:**

Multilevel models showed that moments (Level 1) of higher‐than‐average negative affect (*p* < 0.001) and/or stress (*p* = 0.033) were characterized by less negative emotion differentiation, while moments of higher‐than‐average positive affect were characterized by greater positive emotion differentiation (*p* < 0.001). At the between‐person level (Level 2), participants with higher stress overall had lower negative emotion differentiation (*p* = 0.009). Linear regression showed that average negative, but not positive, emotion differentiation was inversely associated with percent drinking days over the subsequent 3‐month follow‐up period (*p* = 0.042). Neither form of average emotion differentiation was associated with drinking quantity.

**Conclusions:**

We found that for individuals in early AUD recovery, affective states are associated with acute shifts in the capacity for emotion differentiation. Further, we found that average negative emotion differentiation prospectively predicts subsequent alcohol use.

## INTRODUCTION

Deficits in emotion regulation figure prominently in models of alcohol use disorder (AUD) etiology, maintenance, and relapse (Baker et al., 2004; Eddie, Barr, et al., 2021; McCarthy et al., 2010; Sher & Levenson, 1982; Sliedrecht et al., 2019), with many individuals with AUD maladaptively utilizing alcohol to cope emotionally (Lavallo, 2007; Spence & Courbasson, 2012). Impairment in emotion identification (e.g., alexithymia) and processing are also commonly reported in persons with AUD (Breese et al., 2011; Maurage et al., 2011; Monnot et al., 2002; Thorberg et al., 2009). Difficulties identifying emotions may be particularly pronounced in early AUD recovery—a period characterized by heightened negative affect and anhedonia due to the neural and physiological effects of chronic substance use (Eddie et al., 2019; Eddie et al., 2022; Koob, 2009), and acute and chronic alcohol withdrawal syndromes (Breese et al., 2011), as well as heightened stress due to new situational demands (Eddie et al., 2020; Eddie, White, et al., 2021; Sinha, 2012). These experiences are associated with a range of negative outcomes including AUD relapse and poor psychosocial adaptation (e.g., Kornreich et al., 2002; Rupp et al., 2017). It is perhaps not surprising that those who can navigate the affective landscape of early AUD recovery tend to have longer periods of alcohol abstinence (Daughters et al., 2005; Strong et al., 2012). Yet, the mechanisms that might underpin emotional resilience in some moments and in some individuals, but not others, remain unclear.

One mechanism shown to contribute to emotion regulation is emotion differentiation (Feldman Barrett et al., 2001; Tugade et al., 2004). Emotion differentiation is the ability to make nuanced distinctions between similarly valanced emotion states (e.g., sadness vs. jealousy; Feldman Barrett, 2004). Individuals differ greatly in their ability to differentiate their affective experiences, with some making these fine‐grained differentiations easily, and others finding it difficult to make these subtle distinctions, often describing their emotional experiences in global terms such as feeling “good” or “bad.”

It is thought that greater capacity for emotion differentiation helps individuals cope emotionally because differentiation is a necessary first step for employing appropriate and effective emotion regulation strategies. This is because individual emotions have unique sets of antecedents and subsequent strategies to manage them (Gross, 2011). Research on alexithymia, a construct related to deficits in identifying and describing emotions, shows that these deficits increase during depressive episodes (Marchesi et al., 2008), suggesting that large acute shifts in negative affective intensity potentially compromise emotional understanding. Meanwhile, other research shows the inability to differentiate emotion may lead to maladaptive behaviors when emotionally aroused (Emery et al., 2014; Kashdan et al., 2015). Thus, if a person is not able to determine what emotion they are feeling, their ability to effectively problem solve to manage that emotion will be diminished. Given mood repair is often prioritized over achieving other more adaptive long‐term goals (e.g., remaining abstinent; Tice et al., 2001), this commonly leads to “quick‐fix” behaviors aimed at alleviating affective arousal such as risky alcohol use (Kashdan et al., 2010), nonsuicidal self‐injury (Zaki et al., 2013), and physical/verbal aggression (Pond et al., 2012).

Taken together, it follows that poor emotion differentiation in the context of heightened negative affect or stress would contribute to substance use lapses among individuals in early substance use disorder recovery. Supporting this postulate, Anand et al. (2017) showed that emotion differentiation predicts likelihood of initial substance use lapse following residential substance use disorder treatment. This study, however, was not able to discern whether in‐the‐moment negative affect or stress impair one's ability to differentiate negative emotions among those attempting to recover from AUD. This is because until recently emotion differentiation was generally considered a stable individual characteristic and was only able to be measured at the trait level. Newer research, however, shows that emotion differentiation is a dynamic time‐varying process that can be captured at both “the moment” and “trait” levels (Erbas et al., 2021). Yet, to date, no study has examined momentary emotion differentiation in the context of early recovery from AUD or have any studies examined the effects of positive emotion differentiation on lapses to alcohol use, despite evidence suggesting it is protective against alcohol‐related problems (Emery et al., 2014). It is imperative that we understand the distinctive emotional lives of individuals in early recovery to better understand, and by extension, help individuals prepare for the unique challenges of early AUD recovery.

The present study utilized ecological momentary assessment (EMA) to examine if moments of high affective arousal are characterized by differences in emotion differentiation among those in the first year of a current AUD recovery attempt, and if a person's average emotion differentiation measured during the EMA monitoring period would prospectively predict subsequent alcohol use over a 3‐month follow‐up.

It was hypothesized that moments of high negative affect and/or stress would be characterized by decreased momentary positive and negative emotion differentiation, respectively, consistent with an impaired ability to self‐regulate. Based on previous findings (Erbas et al., 2021), we also hypothesized that moments of high positive affect would be characterized by higher emotion differentiation. This finding would be consistent with the “broaden and build” theory (Fredrickson, 2001) that suggests that positive emotions broaden one's awareness and encourage novel, exploratory thoughts and actions, which over time build useful skills and psychological resources. In line with structural models of affect that demonstrate that negative affect, stress, and positive affect are distinct, yet related constructs (Clark & Watson, 1988; Watson & Clark, 1994; Watson & Tellegen, 1985), we opted to model each affective construct separately to evaluate their unique contributions. Finally, we predicted that average positive and negative emotion differentiation would be inversely associated with percent of drinking days (PDD) and drinks per drinking day (DDD) over a 90‐day follow‐up.

## METHODS

### Participants

Participants were recruited from the greater Boston area through Mass General Brigham hospital group's study pool networks, online postings, flyers, handouts, and targeted mail to AUD treatment programs, sober homes, medical facilities, and local psychology departments. Inclusion criteria included: (1) meeting current Diagnostic and Statistical Manual of Mental Disorder 5 (American Psychiatric Association, 2013) AUD criteria (mild, moderate, or severe); (2) endorsing a current goal of alcohol abstinence; (3) being in the first year of a current AUD recovery attempt; and (4) being engaged in outpatient treatment for AUD (e.g., partial hospital programs), mutual‐help programs (e.g., Alcoholics Anonymous), or individual treatment (e.g., therapist). Because the parent study included physiological monitoring, participants were required to have 2 weeks of self‐reported alcohol and other drug abstinence before enrolling in the study to minimize the influence of physiological withdrawal symptoms.

As previously reported (Eddie, Barr, et al., 2021), the sample was 61.9% male (*N* = 42) and ranged from ages 18 to 65 (*M* = 41.6, SD = 12.6). Participants were 73.8% White/European American, 19.1% Black/African American, 4.8% Asian, and 2.4% Other race/Mixed race. Median income was $35,500 (*M* = $56,999.55, SD = $57,536.85, Range = $0 to 230,000). Additionally, participants had a mean of 84.7 (SD = 99.5) days since their last drink at baseline. Three participants were lost to follow‐up.

### Procedure

Study eligibility was determined with a phone screen. Eligible participants completed an intake appointment with baseline measures and were oriented to the EMA application on their smartphone. Participants then completed 6 days of EMA monitoring with both random and self‐initiated EMA surveys using the MetricWire EMA smartphone application (MetricWire, 2016). The program generated three prompts for participants to complete brief ~2‐min assessments about participants’ experience at random times within 3‐h blocks each day (e.g., 10:00 a.m. to 1:00 p.m.). In addition to random surveys, participants were instructed to self‐initiate a survey in moments when they felt high levels of stress, alcohol craving, or felt at risk for alcohol use. EMA surveys included measures of affect and stress as well as descriptive information such as location and social context. To encourage random EMA survey completion, participants were given a $30 bonus for completing ≥90% of the surveys. Finally, 90 days following the end of the 6‐day EMA monitoring period, participants were remotely assessed for past 90‐day alcohol use. This research was approved by Mass General Brigham's institutional review board (IRB# 2016P001178).

### Baseline measures

#### AUD severity

Baseline AUD severity was measured using the Alcohol Dependence Scale (ADS; Skinner & Allen, 1982). The ADS consists of 25 items that inquire about symptoms experienced during the past 90 days. Scores on the ADS range from 0 to 47. According to Skinner and Allen (1982), a score of 1 to 13 represents a low level of AUD (first quartile), 14 to 21 an intermediate level (second quartile), 22 to 30 a represents a substantial level (third quartile), and 31 to 47 a severe level (fourth quartile). The ADS has high levels of internal consistency and obtained a Cronbach's α = 0.90 in this study.

#### Baseline drinking

Timeline follow‐back (TLFB; Sobell & Sobell, 1992) was used to record the number of standard alcoholic drinks consumed over the past 30 days before study intake. The TLFB uses a calendar to help people provide retrospective estimates of their daily drinking over the specified time period. Several memory aids can be used to enhance recall (e.g., calendar; key dates serve as anchors for reporting drinking; standard drink conversion). Baseline drinking was calculated as the PDD in the past 30 days prior to the baseline visit.

### EMA measures

#### Affect

During random and self‐initiated surveys, momentary positive and negative affect were assessed by items from the Positive and Negative Affect Schedule–Expanded Form (Watson & Clark, 1994) and Larsen and Diener's affect circumplex model (Larsen & Diener, 1992). Negative affect was represented by sadness, guilt, nervousness, tiredness, and anger. Positive affect was represented by happiness, calmness, and energy. Participants were instructed to report the intensity of the specific emotions on 11‐point scales ranging from 0 (*none*) to 10 (*extreme*). For the present analyses, measures of momentary positive and negative affect were calculated by taking the mean of the respective positive and negative affect items at each moment (i.e., state). These were aggregated into person‐level averages for each participant and used as a measure of their average level of positive and negative affect (i.e., trait). Previous research supports the criterion validity of these affective items assessed using EMA (Emery et al., 2020; Hoeppner et al., 2014; Kashdan et al., 2010; Muraven et al., 2005).

#### Stress

Momentary perceived stress was measured during random surveys and self‐initiated surveys. Participants were instructed to report their feelings of stress using a 11‐point scale ranging from 0 (*no stress*) to 10 (*extreme stress*). These momentary assessments were then aggregated into a person‐level average for each participant and used as a measure of their average stress level. This single‐item approach is commonly used in EMA research that has demonstrated good criterion validity (e.g., Szeto et al., 2019).

#### Emotion differentiation

Traditionally, emotion differentiation is created as a between‐person variable from EMA data by calculating the intraclass correlation (ICC with absolute agreement) of the positive and negative emotion terms, respectively, for each participant across the momentary assessments (Kashdan et al., 2010; Pond et al., 2012). This calculates the percent of the total variation in emotion ratings due to variation across assessment time points versus variability between emotion terms within time points. This value ranges from 0 to 1 and represents the extent to which same valenced emotions covary. The inverse is used so that higher scores equal greater differentiation between emotions. The criterion validity of this approach is supported by recent research indicating significant associations between this measure and the difficulty identifying feelings subscale of the Toronto Alexithymia Scale—20 (Erbas et al., 2014).

Momentary emotion differentiation is derived directly from and perfectly related to the classic between‐person emotion differentiation index. Momentary emotion differentiation was calculated separately for positive and negative emotions following the procedure of Erbas et al. (2021), using the following steps: (1) each momentary assessment of emotion (e.g., happy, sad) was person‐mean centered making each observation reflect moment‐to‐moment deviations from a person's average level of that specific emotion; (2) the means of the centered emotions for each time point were then calculated; (3) this was then multiplied by the number of emotions and the product squared. This is the numerator of the equation. Next, (4) the variance for each of the centered emotions was computed, and then (5) the sum of the variances of all the emotions was calculated. This is the denominator of the equation. Finally, (6) we computed the equation and multiplied the outcome by −1. This created a series of negative values that includes 0, where larger numbers equal to greater momentary emotion differentiation and smaller numbers are equal to worse momentary emotion differentiation. That is, momentary positive and negative emotion differentiation will be more negative when an individual's emotion differentiation is worse than their overall mean level and closer to 0 when an individual's emotion differentiation is better than their average.

In other words, a more strongly negative value means that, at that specific time point, the level of differentiation is low relative to the individual's overall level of differentiation. In practice, this means that if an individual encounters an emotional situation that results in higher‐than‐average levels of anger, sadness, and anxiety, momentary emotion differentiation will be strongly negative, indicating lower levels of momentary differentiation. However, if they encounter an emotional situation that results in higher‐than‐average levels of anger, but lower‐than‐average levels of sadness and anxiety, then momentary emotion differentiation will be closer to 0, indicating higher levels of differentiation. Momentary emotion differentiation scores were aggregated into person‐level averages for each participant and used as a measure of their average level of positive and negative emotion differentiation.

Importantly, as detailed by Erbas et al. (2021), this momentary index is directly derived from the classic trait emotion differentiation index, the ICC. Averaging all of the momentary indices for each individual results in an index with a perfect nonlinear relationship with the overall ICC. This relationship is monotonically increasing, implying that the Spearman rank correlation between both measures equals 1. This means that the traditionally used between‐person ICC can be reduced to these momentary indices, each of which contribute to the overall ICC.

### Follow‐up measures

#### Drinking goal

At baseline, participants were asked to describe their alcohol use goal. To be included in the study, potential participants needed to endorse an abstinence goal. Participants chose from the following options: *total abstinence (0)*, *moderation (1)*, and *no drinking goal (2)*. Because participants’ alcohol use goal could have changed through the course of the study, alcohol use goal was assessed again at 90‐day follow‐up, with participants asked to endorse their alcohol use goal over the majority of the follow‐up period. Drinking goal assessed at follow‐up was used in our models as a covariate due to its probable association with drinking.

#### Drinking over the follow‐up period

TLFB (Sobell & Sobell, 1992) was used to record the number of standard alcoholic drinks over the 90 days following the end of the 6‐day EMA monitoring period. The 90‐day follow‐up was conducted using online forms and, if necessary, a concurrent phone call. Research shows that the TLFB provides accurate measurements of alcohol consumption over the phone and online (Pedersen et al., 2012; Sobell & Sobell, 1992). Two drinking indices were derived from the follow‐up data: number of drinking days and DDD. Follow‐up percent drinking days (PDD) was calculated as the percentage of days, in the past 90 days, in which alcohol was consumed. Follow‐up DDD was calculated as the number of drinks consumed over the follow‐up period divided by the number of drinking days in the past 90 days.

### Transparency and openness

We report below how we determined our sample size and all data exclusions. Data were analyzed using Stata 15 (StataCorp, 2017). Stata code to calculate emotion differentiation metrics can be found at https://osf.io/wymnd/. This study's design and its analysis were not preregistered.

### Analysis plan and minimum detectable effect size

To test the effects of affective state on momentary emotion differentiation during the EMA monitoring period, we estimated multilevel models with random intercepts and with an unstructured variance–covariance matrix using Stata 15 (StataCorp, 2017). The data had a two‐level structure in which moments (Level 1 [L1]; within‐person) were nested within persons (Level 2 [L2]; between persons). Multilevel models account for the nonindependence of observations that results from the nesting of time‐varying observations via momentary random assessments within persons (L1). The models contained within‐person stress, negative affect, and positive affect predicting emotion differentiation at the same moment. Separate models for positive and negative emotion differentiation were estimated.

Level 1 focal predictors were within‐person stress, positive affect, and negative affect. In addition to the focal predictors at L1, six orthogonal day‐of‐the‐week indicators and day in the study were included as covariates. The inclusion of day of the week addresses daily variation in mood and reduces potential serial auto‐correlation across days (Mohr et al., 2001). Inclusion of the number of days since initiating the study adjusts for change over time not associated with the focal predictors. L2 focal predictors were between‐person aggregates of the momentary affect and stress indicators. L1 variables were centered within‐persons by subtracting person averages from momentary values (i.e., person‐centered). L2 variables were centered by subtracting the overall sample averages from person‐level averages (grand‐mean centered). In this context, person‐centered variables reflect moment‐to‐moment deviations from a person's average level, and grand‐mean centered variables reflect person deviations from the overall average for the sample.

Distinctions between person‐average affect (e.g., dispositional positive affect) and stress (i.e., dispositional stress) from momentary states (e.g., a moment of high positive affect and/or stress) capitalized on the full complement of the EMA data by distinguishing dynamic, within‐person changes in momentary affect or stress from between‐person, individual differences in typical (i.e., dispositional) affect or stress level. Importantly, EMA data also provide the advantage of temporal specificity. Affect and stress were expected to vary not only within each person over time (L1; within person) but also on average from person to person (L2; between persons). The inclusion of momentary and average indicators allowed for isolation of the within‐person associations of affect and stress fluctuations with emotion differentiation from between‐person associations. An intercept‐only model (without any predictors) estimated intraclass correlations (i.e., variability in predictors attributed to between‐person effects relative to within‐person influences).

To test the hypothesis that positive and negative emotion differentiation would be associated with alcohol use over the follow‐up period, we estimated a linear regression model in Stata 15 (StataCorp, 2017) where person‐level average positive and negative emotion differentiation derived from the EMA data predicted PDD during the 3‐month follow‐up, while controlling for drinking goal (moderation or abstinence), sex, race, and age, given these are likely sources of differences in drinking.^1^ For instance, those that opted for a moderation goal are likely to report more drinking days than those that opted for an abstinence goal. Predictors were centered at the sample mean (i.e., grand‐mean) and robust standard errors were calculated using Huber–White sandwich estimators to accommodate for heteroscedasticity (Croux et al., 2004). We did not correct for multiple comparisons in our models following the guidance of Rothman (1990). Instead, describing what tests of significance were performed, and why, is suggested as best practice because corrections protect against Type I errors, but also risk creating Type II errors in which important differences might be deemed nonsignificant (Leek et al., 2017; Rothman, 1990).

To determine the minimum detectable effect size from the number of observations in our data, we conducted a computer simulation using the Monte Carlo feature of Mplus 8.5 (Muthén & Muthén, 2017). Consistent with previous research, 50% of the variance in affect and stress was specified at the within‐person level with the remaining variance specified at the between‐person level (e.g., Emery & Simons, 2020). The focal effects of interest in this study are the effects of momentary affect/stress on momentary emotion differentiation. Unfortunately, previous research has not examined these associations in this population. Thus, we conducted multiple Monte Carlo simulations with small effect sizes ranging *β* = 0.05 to *β* = 0.35 for both within‐ and between‐level associations. Results using 10,000 replications indicated a sample of 42 individuals with 20 observations each would be sufficiently powered to detect within‐person effects of *β* = 0.07 or higher, and between‐person effects of *β* = 0.33 or higher. Importantly, the final models will have more observations as well as a series of covariates, which will account for additional residual variance not estimated here, effectively increasing power above what was seen here. Accordingly, based on these simulations, we appear to be adequately powered to detect any clinically meaningful effects found in these models.

## RESULTS

### Descriptive statistics

Compliance with random surveys was calculated by dividing the total number of random surveys completed by all participants by the total number completed plus missed random surveys. There were 684 completed random surveys and 756 combined completed + missed random surveys; thus, compliance was 90.5%. On average, participants completed 16.3 (SD = 5.1) random EMA surveys and 5.5 (SD = 6.4) self‐initiated EMA surveys over the 6‐day monitoring period. Participants’ mean ADS score was 24.2 (SD = 8.8), indicating a “substantial level” of AUD severity (Skinner & Horn, 1984). The sample had on average 12.0 PDD (SD = 18.5) and 3.6 (SD = 5.7) DDD over the 30 days immediately preceding study enrollment. Over the 90‐day follow‐up period, participants averaged 11.1 (SD = 20.5) PDD and 3.9 (SD = 7.5) DDD, with 84.6% endorsing a goal of alcohol abstinence over the follow‐up period, and 15.4% endorsing a goal of alcohol use moderation. Descriptive statistics for Level 1 and Level 2 variables are presented in Table 1.

**TABLE 1:** Descriptive statistics

Variable	*M*	(SD)
Within person (L1; time varying)		
Negative affect	2.52	1.80
Stress	3.25	2.67
Positive affect	5.43	1.89
Negative emotion differentiation	−2.07	3.69
Positive emotion differentiation	−1.49	2.33
Between persons (L2; time invariant)		
Negative affect	2.60	1.57
Stress	3.30	2.09
Positive affect	5.42	1.54
Negative emotion differentiation	−2.08	1.55
Positive emotion differentiation	−1.46	0.80
Percent drinking days	11.08	20.50
Drinks per drinking day	3.91	7.50

Note

*N* = 42. Level 1 observations = 915. *M* = mean; SD = standard deviation. Percent drinking days = percent of days over 90‐day follow‐up period where drinking took place. Drinks per drinking day = average number of drinks per drinking day over 90‐day follow‐up period. L1 variables are “states” varying within‐person across time and L2 are dispositional characteristics aggregated from multiple state assessments (i.e., “traits”).

Stress, positive affect, and negative affect varied across persons and moments. The intraclass correlations were 0.56 for stress, 0.56 positive affect, and 0.66 for negative affect. This indicates that 56% of the variance in stress and positive affect was due to between‐person factors and the remaining 44% was due to moment‐to‐moment within‐person fluctuations. For negative affect, 66% of the variance was due to between‐person differences, whereas 34% was due to within‐person moment‐to‐moment variability.

Not surprisingly, positive and negative emotion differentiation varied between and within persons as well but exhibited a pattern favoring within‐person fluctuations. Specifically, the intraclass correlations were 0.08 for positive emotion differentiation, and 0.14 for negative emotion differentiation. This denotes that between 92% and 86% of the variance in emotion differentiation was at the within‐person level. This finding supports the stance that emotion differentiation is a time varying construct appropriate for event‐level methods and analysis.

### Multilevel model analyses

Multilevel models were estimated to test the hypothesized momentary effects of stress, positive affect, and negative affect on positive and negative emotion differentiation. Separate models were estimated for positive and negative emotion differentiation. It was hypothesized that moments of decreased positive and negative emotion differentiation, respectively, would be characterized by high negative affect and/or stress and moments of high positive and negative emotion differentiation would be characterized by high positive affect.

#### Negative emotion differentiation

Consistent with our hypotheses, at the within‐person level (L1), individuals experienced lower negative emotion differentiation in moments characterized by greater negative affect (*b* = −0.62, *p* < 0.001, 95% CI = −0.88, −0.36) and greater stress (*b* = −0.16, *p* = 0.033, 95% CI = −0.30, −0.01). Contrary to expectations, however, momentary positive affect was not related to momentary negative emotion differentiation (*b* = −0.07, *p* = 0.475, 95% CI = −0.27, 0.13). At the between‐person level (L2), average stress was associated with decreased ability to differentiate negative emotions, on average (*b* = −0.50, *p* = 0.009, 95% CI = −0.87, −0.12). Average negative affect (*b* = 0.02, *p* = 0.943, 95% CI = −0.63, 0.51) and average positive affect (*b* = −0.02, *p* = 0.932, 95% CI = −0.43, 0.39) were both unrelated to a person's average negative emotion differentiation. Sex (*b* = −0.66, *p* = 0.124, 95% CI = −1.50, 0.18), race (*b* = 0.20, *p* = 0.191, 95% CI = −0.10, 0.49), and age (*b* = −0.01, *p* = 0.846, 95% CI = −0.04, 0.03) were not related to a person's average negative emotion differentiation. See Table 2 for full model estimates.

**TABLE 2:** Multilevel models of emotion differentiation

Variable	Negative emotion differentiation model			Positive emotion differentiation model		
	*b*	*SE*	*p*‐value	*b*	*SE*	*p*‐value
Within person (L1; time varying)						
Negative affect	**−0.62**	**0.132**	**<0.001**	0.05	0.086	0.528
Positive affect	−0.07	0.101	0.475	**0.37**	**0.066**	**<0.001**
Stress	**−0.16**	**0.073**	**0.033**	0.01	0.048	0.953
Monday	−0.34	0.434	0.438	0.24	0.282	0.397
Tuesday	0.11	0.435	0.801	0.18	0.282	0.529
Wednesday	−0.75	0.430	0.080	0.05	0.280	0.864
Thursday	−0.36	0.435	0.411	0.20	0.283	0.471
Friday	−0.33	0.435	0.442	−0.08	0.283	0.773
Saturday	−0.12	0.427	0.784	−0.28	0.277	0.320
Day in the study	0.11	0.059	0.064	0.06	0.038	0.106
Between persons (L2; time invariant)						
Negative affect	0.02	0.291	0.943	0.19	0.167	0.247
Positive affect	−0.02	0.210	0.932	−0.13	0.120	0.295
Stress	**−0.50**	**0.190**	**0.009**	−0.11	0.109	0.296
Age	−0.01	0.018	0.918	**0.03**	**0.010**	**0.003**
Race	0.20	0.150	0.191	−0.12	0.086	0.160
Sex	−0.66	0.428	0.124	−0.08	0.244	0.759

Note

*N* = 42. Level 1 observations = 915. Sex = sex assigned at birth (1 = male, 0 = female). Race = racial identity (0 = White). *b* = unstandardized coefficients. *SE* = standard error. Level 1 variables were person centered and Level 2 variables were grand‐mean centered. Sunday was the reference group for day of the week indicators.

#### Positive emotion differentiation

At the within‐person level (L1), individuals experienced higher positive emotion differentiation in moments characterized by greater positive affect (*b* = 0.37, *p* < 0.001, 95% CI = 0.24, 0.50) as hypothesized. However, contrary to hypothesis, both momentary negative affect (*b* = 0.05, *p* = 0.528, 95% CI = −0.11, 0.22) and momentary stress (*b* = 0.01, *p* = 0.953, 95% CI = −0.09, 0.10) were unrelated to momentary positive emotion differentiation. At the between‐person level (L2), average stress (*b* = −0.11, *p* = 0.296, 95% CI = −0.33, 0.10), average negative affect (*b* = 0.19, *p* = 0.247, 95% CI = −0.13, 0.52), and positive affect (*b* = −0.13, *p* = 0.295, 95% CI = −0.36, 0.11), race (*b* = −0.12, *p* = 0.160, 95% CI = −0.29, 0.05), and sex (*b* = −0.08, *p* = 0.759, 95% CI = −0.55, 0.40) were not related to a person's average positive emotion differentiation. However, age was significantly associated with average positive emotion differentiation (*b* = 0.03, *p* = 0.003, 95% CI = 0.01, 0.05). See Table 2 for full model estimates.

### Regression analyses

A pair of linear regression models were estimated to test the hypothesized, prospective effect of average positive and negative emotion differentiation on alcohol use over follow‐up. It was hypothesized that average positive and negative emotion differentiation would be inversely associated with both the PDD (i.e., drinking frequency) and the average DDD (i.e., drinking quantity) over the 3‐month follow‐up period.

#### Frequency model

Consistent with our hypothesis, average negative emotion differentiation exhibited an inverse prospective relationship with drinking frequency over the 3‐month follow‐up (*β* = −0.25, *p* = 0.042). However, inconsistent with our hypothesis, average positive emotion differentiation was not related to drinking frequency over the follow‐up (*β* = −0.14, *p* = 0.265). See Table 3 for full model estimates *F* (6, 32) = 4.74, *p* = 0.002, *R* ^2^ = 0.64.

**TABLE 3:** Regression model of percent drinking days

Variable	*β*	Robust *SE*	*p*‐value
Drinking goal	**0.72**	**0.10**	**0.001**
Age	0.16	0.01	0.169
Race	0.03	0.01	0.694
Sex	0.18	0.05	0.108
Positive emotion differentiation	0.14	0.03	0.265
Negative emotion differentiation	**−0.25**	**0.02**	**0.042**

Note

*N* = 39. Drinking goal = drinking goal over 90‐day follow‐up period (0 = abstinence, 1 = moderation). Sex = sex assigned at birth (1 = male, 0 = female). Race = racial identity (0 = White). *β* = standardized beta coefficient. *SE* = standard error. Positive emotion differentiation = person‐mean aggregate. Negative emotion differentiation = person‐mean aggregate.

#### Quantity model

In contrast to our hypotheses, neither average negative emotion differentiation (*β* = 0.15, *p* = 0.281) nor average positive emotion differentiation (*β* = 0.13, *p* = 0.356) were related to drinking quantity over the 3‐month follow‐up. See Table 4 for full model estimates *F* (6, 32) = 0.72, *p* = 0.639, *R* ^2^ = 0.09.

**TABLE 4:** Regression model of drinks per drinking day

Variable	*β*	Robust *SE*	*p*‐value
Drinking goal	−0.09	2.19	0.408
Age	0.01	0.07	0.947
Race	−0.06	0.46	0.517
Sex	−0.12	2.22	0.440
Positive emotion differentiation	0.13	1.32	0.356
Negative emotion differentiation	0.15	0.76	0.281

Note

*N* = 39. Drinking goal = drinking goal over 90‐day follow‐up period (0 = abstinence, 1 = moderation). Sex = sex assigned at birth (1 = male, 0 = female). Race = racial identity (0 = White). *β* = standardized beta coefficient. *SE* = standard error. Positive emotion differentiation = person‐mean aggregate. Negative emotion differentiation = person‐mean aggregate.

## DISCUSSION

Informed by contemporary models of AUD relapse that posit that problems self‐regulating affect are central to resumption of alcohol use in early AUD recovery (Sliedrecht et al., 2019; Witkiewitz & Marlatt, 2004), we examined within‐ and between‐person associations among positive and negative affect and emotion differentiation. Our goal was to determine if in‐the‐moment negative affect and/or stress impairs one's ability to differentiate negative emotions, and if in the moment positive affect facilitated the ability to differentiate positive emotions, among those in early recovery from AUD. Building on evidence that emotion differentiation is a protective factor against relapse and alcohol‐related problems (Anand et al., 2017; Emery et al., 2014), we also examined if average emotion differentiation would prospectively predict drinking outcomes.

Our findings add to a small but growing body of literature on the protective effects of negative emotion differentiation on return to substance use among those in recovery from substance use disorders. Results also underscore the dynamic nature of emotion differentiation and show that increased negative affect and stress—both hallmarks of early AUD recovery—on average, make it more difficult for individuals to differentiate negative emotions in the moment. Meanwhile, increased positive affect was associated with an increased ability to differentiate positive emotions. In the following sections, these findings are discussed with respect to the theoretical rationale that formed our hypotheses.

### Emotion differentiation and alcohol use

It is thought that one's capacity for emotion differentiation influences their ability to self‐regulate affect because the ability to distinguish between emotional states supports employment of better fitting affect regulation and coping strategies (Kashdan et al., 2010, 2015; Tamir, 2009). This is particularly relevant to individuals in early AUD recovery who are prone to experiencing stress and emotional vulnerability (Eddie et al., 2020; Sher & Grekin, 2007; Sinha, 2012), which are potent risk factors for AUD relapse. Our results suggest that individuals with high emotion differentiation, on average (i.e., dispositional), may be well equipped to achieve recovery goals during this emotionally vulnerable transition period. Specifically, we found that average negative emotion differentiation was inversely associated with PDD, such that those with higher average negative emotion differentiation drank less frequently over 90 days following a 6‐day EMA monitoring period, and those with lower average negative emotion differentiation reported higher percentage of drinking days. However, this pattern appeared to be contained to frequency of drinking given there were no prospective effects for emotion differentiation of either affective valence on drinking quantity (i.e., DDD).

This former finding is consistent with Anand et al. (2017), who measured affect five times over the course of 12 months and found that negative emotion differentiation predicted lower likelihood of initial substance use lapse 1‐year posttreatment. We extend their findings by deriving a measure of trait negative emotion differentiation from 6 consecutive days of affect ratings. This is in line with research that shows EMA methods can be used to collect multiple‐state assessments and aggregate them to make reliable dispositional measures of complex phenomenon (Emery et al., 2020; Emery & Simons, 2020). Additionally, Anand et al. found cross‐sectional associations between emotion differentiation and substance use lapses, although for most participants, affect ratings used to calculate emotion differentiation were obtained after their initial lapse.

Our findings extend this work by finding a prospective relationship between negative emotion differentiation and alcohol use in early recovery. Studies have shown that the ability to regulate and tolerate negative affect predicts lower levels of substance use (e.g., Strong et al., 2012). Also consistent with our results, Kashdan et al. (2010) found that when individuals were stressed immediately prior to drinking, dispositional negative emotion differentiation attenuated consumption. Our results extend these findings by showing that dispositional negative emotion differentiation is associated with alcohol use over a 90‐day period, a stressful and emotionally vulnerable time (Sher & Grekin, 2007; Sinha, 2012).

### Emotion differentiation, affect, and stress

From a treatment perspective, it is important to understand the unique emotional challenges faced by individuals in early AUD recovery. To our knowledge, this is the first study to examine momentary emotion differentiation, affect, and stress in the context of early recovery from any substance use disorder. The stress inherent in early addiction recovery may contribute to deficits in emotion differentiation. Indeed, as we hypothesized, and consistent with Erbas et al. (2022), at the within‐person level, moments in which individuals experienced higher than average negative affect and stress were associated with moments of lower than average negative emotion differentiation.

This is consistent with the dynamic model of affect (Zautra et al., 2001), which posits that during stressful moments, the magnitude of associations between emotions increases, putatively affording a simpler representation of the situation. Individuals in early AUD recovery face stressful challenges related to, for example, dealing with cravings to use substances and navigating novel socio‐environmental contexts. The impact of these stressors on emotion regulation may play a fundamental role in determining long‐term treatment outcomes. While the stresses of early recovery may be inevitable, there is evidence that emotion differentiation is a modifiable construct (Kircanski et al., 2012; Van der Gucht et al., 2019) that could be an important treatment target during and after substance use treatment.

At the between‐person level, stress and both positive and negative emotion differentiation were also inversely associated, which is consistent with Erbas et al. (2018), and suggests that those who experience greater stress on average find it more challenging to determine exactly what they are feeling. This has clinical implications for relapse prevention, highlighting the importance of helping patients manage stress. However, at the between‐person level, there was no association between negative affect and negative emotion differentiation.

As hypothesized, positive affect was positively associated with positive emotion differentiation at the within‐person level. These results reinforce research demonstrating positive affect and positive coping mutually build on one another (Burns et al., 2008). This is consistent w**i**th the “broaden and build” theory (Fredrickson, 2001), which suggests that positive emotions expand one's momentary cognitive‐behavioral repertoire to foster creative and adaptive self‐regulation. This effect may be particularly important for individuals in early recovery from AUD who may have limited regulatory strategies, which could leave them liable to return to dysregulated alcohol use. Also, we observed an association between age and average positive emotion differentiation such that older individuals exhibited better positive emotion differentiation which is consistent with previous work suggesting emotion differentiation is a skill that is acquired throughout the lifespan (Nook et al., 2018).

Contrary to hypothesis, neither momentary stress nor momentary negative affect was associated with momentary positive emotion differentiation. Taken together, this pattern of results underscores the importance of identifying aspects of affect aside from level of arousal that may be useful for clarifying the role of affective functioning in alcohol use at the within‐person level. This study thus represents a novel approach to understanding the links between affective functioning and alcohol use. This is especially true given Anand et al. (2017) did not find a cross‐sectional association between average affective intensity and dispositional negative emotion differentiation.

### Clinical implications

There are several aspects of the current study with important clinical implications. The pattern of results indicates that specific deficits in emotional identification are a critical issue complicating the emotional lives of those in early recovery from AUD that appears to have downstream effects on alcohol use patterns. Emotion differentiation is best conceptualized as a skill lying on a continuum of risk to resilience for maladaptive behavioral outcomes in emotionally at‐risk individuals, such as those with AUD. We showed here that heightened negative affect and stress are associated with a diminished capacity for emotion differentiation. While some negative feelings are of course unavoidable, this work suggests interventions that promote emotion regulation as well as better emotion differentiation have potential to help individuals in early AUD recovery to more effectively navigate high‐risk situations.

Similarly, our finding indicating that positive emotions support positive emotion differentiation also has clinical relevance. If increasing positive affect is related to better emotion differentiation then treatments designed to increase positive affect (rather than exclusively focusing on mitigating negative affect; e.g., Daughters et al., 2018), it might be particularly helpful at facilitating emotional well‐being (i.e., upward spiral). Taken together, there are clear costs associated with reduced capacity for emotion differentiation, as well as benefits associated with emotion differentiation proficiency. As such, emotion literacy programs that focus on positive and negative emotion identification, links between antecedents and emotional consequences, and the natural time course of emotional arousal might also be useful adjuncts to current treatment practices.

### Strengths and limitations

Several limitations of the current study should be noted. Although our analytic approach was rigorous, we disaggregated within‐ and between‐person effects, and we controlled for contextual factors of import to longitudinal data, these data were from a proof‐of‐concept study with a small sample size and short EMA monitoring period. This precluded testing more complex models.

Previous research indicates emotion labeling is compromised by intense mood states, such as a depressive episode, and abates as affective arousal decreases, but emotion labeling does not prospectively predict shifts in mood (Marchesi et al., 2008). This supports the hypothesized model whereby dynamic shifts in emotional intensity lead to acute changes in emotion differentiation rather than vice versa. However, further research is needed to examine whether reflective processes underlying the labeling of feeling states impact affective arousal.

Some investigators have questioned the validity of using an ICC‐derived index of emotion differentiation (e.g., Thompson et al., 2021). Conceptually, one may experience and differentiate intense levels of, for example, anger, sadness, and guilt at the same moment. Statistically, however, they may not be considered as differentiating at all because the emotions perfectly covary (e.g., they are all rated as 10—*extreme*). While we recognize this as a limitation of the ICC‐derived index of differentiation, signals with no variability in the same valenced emotion ratings were rare in our sample (i.e., 6% of signals). Thus, we believe our metric of differentiation is statistically capturing most of what it is meant to, conceptually. Furthermore, expected associations among ICC‐based differentiation and alexithymia (Erbas et al., 2014), standardized lab tasks of emotion differentiation (Erbas et al., 2019), and self‐reported emotion valence focus (Erbas et al., 2015) support the validity of our approach.

Additionally, the inclusion criteria required 2 weeks of abstinence because acute withdrawal symptoms could impact a physiological monitoring aspect of the parent study. Accordingly, our results might not generalize to those in acute withdrawal. It is possible these findings might be stronger in such a sample, but this is a question that requires further study. Also, there was relatively limited dimensionality of positive emotions assessed. This could have impacted the results in unknown ways.

There is research suggesting emotion differentiation increases with intensive EMA self‐monitoring (i.e., 8 or more surveys per day over several weeks; Hoemann et al., 2021). However, “day in the study” was included as a predictor of emotion differentiation in the models presented here and its nonsignificance suggests that there was no learning effect observed in our data. This difference may be due to the relatively low number of days and prompts per day in our protocol. Also, its inclusion means any observed effect in our models would be significant after controlling for any learning that might have taken place.

The current study did not include a measure of alcohol use in the EMA battery, or was alcohol assessed retrospectively for the EMA monitoring period. As a result, we were not able to test the full momentary cascade of affect/stress to emotion differentiation to alcohol use. Lastly, a subset of participants (*n* = 6) changed their goal from abstinence to moderation over the 90‐day follow‐up period which, not surprisingly, was associated with greater drinking over follow‐up. Although statistically controlled for, this could have influenced the results in unknown ways.

## CONCLUSIONS AND FUTURE DIRECTIONS

This is the first study to examine if moments of high affective arousal are characterized by differences in emotion differentiation among those in early AUD recovery, and if a person's average emotion differentiation measured during an EMA monitoring period would predict subsequent alcohol use. Results showed that moments of higher‐than‐average negative affect and/or stress were characterized by less negative emotion differentiation, while moments of higher‐than‐average positive affect were characterized by greater positive emotion differentiation. Additionally, those with higher average stress levels had lower negative emotion differentiation, and those with greater average negative emotion differentiation had less PDD over 90‐day follow‐up. Findings suggest that for individuals in early AUD recovery, affective states are associated with the capacity for emotion differentiation, and emotion differentiation influences subsequent alcohol use. Future studies should test the full momentary cascade of affect/stress to emotion differentiation to alcohol use. Relatedly, future studies examining other complex affective dynamics (e.g., affective bipolarity, emotional complexity) or drinking motives could provide important insights into the unique challenges faced by individuals in early AUD recovery.

## CONFLICT OF INTEREST

No conflicts of interests.

## ENDNOTE

^1^
An alternative model that included person‐average negative affect, stress, and positive affect as predictors did not result in an improved model Δ*χ* ^2^ (3, *N* = 39) = 0.59, *p* = .900, and none of these new predictors were significant (negative affect, *p* = .869; stress, *p* = .589; positive affect, *p* = .721). Thus, we opted for the most parsimonious model given our small sample size.
